# Concerns, quality of life, access to care and productivity of the general population during the first 8 weeks of the coronavirus lockdown in Belgium and the Netherlands

**DOI:** 10.1186/s12913-021-06240-7

**Published:** 2021-03-12

**Authors:** Hanne van Ballegooijen, Lucas Goossens, Ralph H. Bruin, Renée Michels, Marieke Krol

**Affiliations:** 1 IQVIA, Herikerbergweg 314, 1101 CT Amsterdam, The Netherlands; 2 Erasmus University Rotterdam, Erasmus School of Health Policy & Management, Rotterdam, The Netherlands

**Keywords:** Coronavirus, COVID-19, Population survey, Quality of life, Productivity, Medical resource use, Burden of disease

## Abstract

**Background:**

The COVID-19 pandemic has a disruptive impact on our society. We therefore conducted a population survey to describe: 1) stress, concerns and quality of life 2) access to healthcare and cancelled/delayed healthcare and 3) productivity during the first 8 weeks of the coronavirus lockdown in the general population.

**Methods:**

An online cross-sectional survey was conducted in a representative sample after 8 weeks of the coronavirus lockdown in Belgium and the Netherlands. The survey included a series of three validated questionnaires about quality of life delayed/cancelled medical care and productivity loss using validated questionnaires.

**Results:**

In total, 2099 Belgian and 2058 Dutch respondents completed the survey with a mean age of 46.4 and 42.0 years, respectively. Half of the respondents were female in both countries. A small proportion tested positive for COVID-19, 1.4% vs 4.7%, respectively. The majority of respondents with a medical condition was worried about their current health state due to the pandemic (53%) vs (63%), respectively. Respondents experienced postponed/cancelled care (26%) and were concerned about the availability of medication (32%) for both countries. Productivity losses due to the COVID-19 restrictions were calculated in absenteeism (36%) and presenteeism (30%) for Belgium, and (19%) and (35%) for the Netherlands. Most concerns and productivity losses were reported by respondents with children < 12 years, respondents aged 18–35 and respondents with an (expected) COVID-19 infection.

**Conclusions:**

This study describes stress, quality of life, medical resource loss and productivity losses in Belgium and the Netherlands after 8 weeks of coronavirus lockdown. The results underline the burden on society.

**Supplementary Information:**

The online version contains supplementary material available at 10.1186/s12913-021-06240-7.

## Background

The COVID-19 pandemic has had an enormous disruptive impact on societies throughout the world [1, 2], not only because of its direct impact on patients with COVID-19, but also due to fear and stress [3]. These include fears for unemployment due to COVID-19-related restrictions, financial worries and concerns about one’s own health. These stressors and worries may be reflected in lower quality of life.

Next to the impact of the COVID-19 related restrictions on quality of life, concerns and stress, the COVID-19 pandemic also caused a substantial strain on the healthcare system [4]. To cope with the COVID-19 infected patients, healthcare facilities had to cancel regular care. In addition, patients may have been anxious to visit their physician or general practitioner because of fear of infection or to avoid further burdening the healthcare system. This could lead to large, secondary healthcare problems such as lower number of diagnoses of critical diseases and exacerbation of existing health conditions without medical intervention.

Additionally, the COVID-19 pandemic has had a wider impact on social and economic factors [5, 6]. Governments have imposed measures aimed at reducing the spread of the virus. These changes imposed on society impacted individuals and organizations. As a result, many individuals and organizations were forced to change their normal routines. Examples are closing of schools and day-care centers, restaurants and sport centers, and asking people to work from home. Many individuals who needed to work from home were forced to combine work with childcare. These measures may have impacted individuals’ productivity, as people may not have been able to work at their normal capacity. Not being able to work at normal capacity while at work (either at home or in the office) is referred to as presenteeism. Others may not have been able to work at all, for instance those working in restaurants or sport centers. Not being able to do one’s work at all is referred to as absenteeism.

Although the combination of presenteeism and absenteeism may have had a widespread impact on society, there is little scientific information on these effects in the general population. Therefore, the aim of this study was to observe and record the developments during the coronavirus lockdown in Belgium and the Netherlands. The governments of Belgium and the Netherlands imposed a lockdown in early March 2020 to prevent further spread of the coronavirus. In Belgium, the recommended distance was 1.5 m. In addition, it was forbidden to go outside if not strictly necessary and schools and universities were closed [7]. In the Netherlands, individuals could leave their home, but were asked to stay at home as much as possible. They also needed to keep 1.5 m distance. Schools, day-care facilities, restaurants and sport centers were closed in both countries.

The aim of this research is to conduct a population survey to describe: 1) stress, concerns and quality of life 2) access to healthcare and cancelled/delayed healthcare and 3) productivity during the first 8 weeks of the coronavirus lockdown in two general populations in Belgium and the Netherlands.

## Methods

### Study design and participants

A cross-sectional, web-based survey was conducted in Belgium and the Netherlands in the beginning of May 2020. At that time, both Belgium and the Netherlands had experienced 8 weeks of COVID-19-related restrictions. Data were collected over a time span of 1-week in a large online panel by sampling agency Dynata. The aim was to include a sample of 2000 respondents from both countries of 18 years and older with a representative spread for age, sex, education and region. The Belgian sample consisted of equal proportions of Dutch and French speakers. Since the questionnaire was programmed electronically, respondents could only finish the questionnaire by completing all questions and thereby missing data was not present.

The data collection was in accordance with local and European privacy legislation. Respondents’ identity remained unknown to researchers and all respondents provided informed consent regarding their participation in the survey and for scientific publication by means of the electronic questionnaire. The study did not include medical records or human tissue; therefore, this study does not require ethical approval and was therefore waived by the national regulations – Central Committee on Research involving human Subjects (CCMO) [8]. Respondents that were younger than 18 years, failed to provide electronic informed consent or refused that their data would be used for scientific research were excluded.

### Questionnaire

The questionnaire consisted of a series of three validated questionnaires about quality of life, cancelled/delayed medical care, and productivity loss. The questionnaire and was developed in Dutch and translated into French [9–11]. The questionnaire was pilot tested among 5 participants and after a soft launch involving 100 respondents’, small adjustments were implemented to improve the answer categories. Quality of life was assessed by 2 instruments. First, a visual analogue scale was used to evaluate the overall health status of the participants on a scale from 0 to 100. The EuroQol 5 Dimensions questionnaire [12] measures health related quality of life on 5 dimensions (EQ-5D) that map each dimension on a scale from below zero to 1. On that scale zero represents death, 1 represents perfect health and scores below zero reflect health states considered worse than death [9]. The health state values are suitable for use in health economic evaluations [13]. For the calculations, the Tobit with constraints model 3 was used with a constant of 0.953 [13]. Respondents evaluated their health on the day of the questionnaire and before the lock-down. For both quality of life questionnaires respondents were asked to rate their health on the day of the questionnaire as well as the period before the lockdown.

Respondents answered questions related to medical resource use, cancelled or delayed health care appointments due to COVID-19 or COVID-19 related restrictions as assessed by the Medical Consumption Questionnaire (iMCQ) [11]. This instrument was designed to measure medical resource use and includes questions related to the number of visits to various healthcare providers. Questions were adapted to be able to capture missed appointments to healthcare providers, including outpatient or primary care and inpatient or specialist care.

Productivity losses related to COVID-19 and COVID-19 related restrictions were recorded by the iMTA Productivity Cost Questionnaire (iPCQ) [10]. The questions were slightly adapted to reflect the COVID-19 lockdown situation and included questions about employment status, absenteeism and presenteeism. The sample size of our study was based on previous research [13] (*n* > 1000). We aimed for a doubled sample size of the validated questionnaires to account for the added questions related to stress and concerns to provide representative estimates on population level for both Belgium and the Netherlands.

### Statistical analyses

The data were analyzed in IBM SPSS Statistics and summarized in proportions and means with 95% confidence intervals, and means with standard deviations when appropriate. The analyses were performed separately for Belgium and the Netherlands. In addition, subgroups of respondents were created based on age 18–35 years, 35–66 years (for Belgium) and 35–67 (for the Netherlands), and above pension age ≥ 66/≥67 years, respectively. The pension age was 66 years for Belgium and 67 years for the Netherlands. We also categorized both countries by education level (low, middle, high), whereby low includes primary school, lower vocational education, preparatory secondary vocational education, middle includes higher general secondary education, secondary vocational education and high includes higher professional education, and university). Further subgroups were created by COVID-19 infection status including suspected of COVID-19 infection vs or not-infected); and by parent status (people with and without children below 12 years of age).

We calculated productivity costs related to lost paid work due to COVID-19 by multiplying the number of hours lost with the average age-related hourly income in the Netherlands [14] or Belgium [15]. All costs are presented as weekly costs.

## Results

### Study population

A total of 2099 respondents completed the questionnaire in Belgium and 2058 in the Netherlands. The mean age was 46.4 year for Belgium and 47.6 year for the Netherlands (Table 1). Half of the respondents were women in both countries. In Belgium, 1.4% of the respondents tested positive by a confirmed COVID-19 test while this was 4.7% for the Netherlands. In both countries, similar proportions suspected that they might have been infected, without a confirmed test result (27% in Belgium and 22% in the Netherlands).

**Table 1:** Population characteristics by country

	Belgium (***N*** = 2099)	the Netherlands (***N*** = 2058)
Age, year (SD)	46.4 (16.3)	47.7 (16.2)
Female (%)	48.9	50.1
Education level (%)		
Low	29.0	25.6
Middle	50.7	28.6
High	20.3	45.9
Children, yes (%)	57.5	60.7
< 18 years	23.8	29.2
< 12 years	15.5	22.1
< 6 years	9.1	13.5
Tested for COVID-19 infection (%)	6.3	8.8
Tested positive (%)	1.4	4.7
Suspected COVID-19 infection		
Yes/maybe (%)	27.2	21.9

Values are proportions or mean and standard deviation; *SD* standard deviation

### Stress and concerns, quality of life

The results of the questions related to stress and financial concerns are presented in Table 2. A minority in both countries felt stressed (27% in Belgium and 14% in the Netherlands), but the majority reported concern about their personal current and future financial situation (59 and 48% respectively), and even more about the national economies (88 and 86%). Respondents reported good health, whether expressed on a visual analogue scale or by EQ-5D utilities (Table 3). Large proportions were worried about the national COVID-19 measures and about access to healthcare (Table 4).

**Table 2:** Stress and financial concerns during the first 8 weeks of the national COVID-19 measures

	Belgium (*N* = 2099)	the Netherlands (*N* = 2058)
**Stress**		
Extremely	1.7 (0–5.9)	0.8 (0–5.0)
Very much	7.5 (3.4–11.6)	2.7 (0–6.9)
A bit	17.7 (13.8–21.6)	10.9 (6.8–14.9)
No stress	40.9 (37.6–44.2)	32.2 (28.6–35.8)
Not at all	32.2 (28.7–35.7)	53.4 (50.4–56.3)
**Worried about current financial situation**		
Extremely	9.9 (5.8–14.0)	6.9 (2.7–11.0)
Very much	14.8 (10.8–18.8)	10.5 (6.4–14.6)
A bit	34.2 (30.7–37.6)	30.8 (27.2–34.4)
No stress	22.1 (18.3–25.8)	29.3 (25.7–32.9)
Not at all	19.1 (15.32–2.9)	22.5 (18.7–26.3)
**Worried about future financial situation**		
Extremely	9.1 (5.0–13.2)	5.8 (1.6–10.0)
Very much	20.1 (16.3–23.9)	14.4 (10.4–18.4)
A bit	36.4 (33.0–39.8)	36.8 (33.3–40.2)
No stress	20.5 (16.7–24.3)	27.7 (24.0–31.4)
Not at all	13.9 (9.9–17.9)	15.3 (11.3–19.3)
**Worried about national economy**		
Extremely	17.5 (13.6–21.4)	11.9 (7.8–16.0)
Very much	36.4 (32.9–39.8)	30.0 (26.4–33.6)
A bit	33.8 (30.3–37.2)	44.6 (41.4–47.8)
No stress	7.4 (3.3–11.5)	8.9 (40.8–13.0)
Not at all	4.9 (0.7–9.1)	4.7 (0.5–8.9)

Values are percentages with 95% confidence intervals

**Table 3:** Perceived health and quality of life before and during the national COVID-19 measures

	Belgium	the Netherlands
	(*N* = 2099)	(*N* = 2058)
Perceived health during COVID-19	72.9 (71.0–74.0)	73.1 (71.8–75.0)
Perceived health before COVID-19	74.5 (72.6–76.3)	74.8 (72.9–76.7)
EQ-5D during COVID-19 measures	0.79 (0.77–0.81)	0.84 (0.82–0.86)
EQ-5D before COVID-19 measures	0.82 (0.80–0.84)	0.85 (0.83–87)

Values are mean and 95% confidence intervals

**Table 4:** Concerns about care during the first 8 weeks of the national COVID-19 national measures

	Belgium (n = 2099)	the Netherlands (n = 2058)
**Concern about availability of medication**		
Extremely	3.1 (0–8.5)	5.5 (0–11.2)
Very much	6.1 (0.1–11.4)	5.8 (0.1–11.5)
A bit	23.0 (18.2–27.8)	21.2 (16.0–26.4)
No stress	29.6 (25.0–34.2)	38.9 (34.3–43.5)
Not at all	38.2 (33.9–42.5)	28.6 (23.7–33.5)
**Concerns about national COVID-19 measures**		
Extremely	6.8 (0–15.5)	12.8 (3.6–22.0)
Very much	15.7 (7.4–23.9	21.5 (12.8–30.2)
A bit	31.0 (23.5–38.5)	29.0 (20.7–37.3)
No stress	22.9 (15.0–30.8)	21.8 (13.1–30.4)
Not at all	23.6 (15.7–31.5)	15.0 (6.0–24.0)
**Concern about access to care**		
Extremely	5.1 (1.0–9.2)	5.1 (1.0–9.3)
Very much	12.2 (8.2–16.2)	11.7 (7.6–15.8)
A bit	35.4 (32.0–38.8)	34.0 (30.5–37.5)
No stress	24.6 (20.9–28.3)	31.7 (28.1–35.3)
Not at all	22.6 (18.8–26.4)	17.5 (13.6–21.4)

Values are percentages with 95% confidence intervals

### Access to healthcare and cancelled/delayed healthcare

More than a quarter of respondents in both countries had experienced cancellations or postponements of appointments with healthcare providers (Table 5) while a higher percentage of respondents avoided a general practitioner appointment. Most people agreed that they received the care as needed (Table 6). Belgian respondents more often experienced medication supply issues (10.1%) vs the Dutch respondents (15.2%). The youngest age group (18–35 year) reported the highest medication supply issues (13.7%) vs (38.3%).

**Table 5:** Cancelled or postponed health-care by age groups

	Belgium				the Netherlands			
	Total	18–35 year	35–65 year	≥ 66 year	Total	18–35 year	35–66 year	≥ 67 year
N	2099	641	1134	324	2058	560	1175	323
**Experienced cancelled/postponed health-care**	26.3 (24.4–28.2)	28.7% (25.2–32.3)	27.1 (24.5–29.7)	19.1 (14.8–23.4)	26.0 (24.1–27.9)	31.8 (27.9–35.7)	22.4 (20.0–24.8)	19.1 (14.8–23.4)
N	553	184	307	62	535	178	263	94
**Avoided GP appointment**	51.9 (47.7–56.1)	58.2% (51.1–65.3)	51.8 (46.2–57.4)	33.9 (22.1–45.7)	45.0 (40.8–49.2)	62.9 (55.8–70.0)	40.3 (34.4–46.2)	24.5 (14.1–34.9)

Values are percentage with 95% confidence interval

**Table 6:** Issues with access to care and supply of medication by age groups

	Belgium				the Netherlands			
	Total	18–35 year	35–65 year	≥ 66 year	Total	18–35 year	35–66 year	≥ 67 year
N	1265	291	715)	259)	1126	227	646	253
**Experienced medication supply issues**								
	10.1 (8.4–11.8)	13.7 (9.7–17.7)	10.6 (8.3–12.9)	4.6 (2.0–7.2)	15.2 (13.1–17.3)	38.3 (32.0–44.6)	10.8 (8.4–13.2)	5.5 (2.7–8.3)
N	2099	641	1134	324	2058	560	1175	323
**Received care as needed**								
Agree	38.0 (34.6–41.4)	35.9 (29.7–42.1)	37.4 (32.8–42.0)	44.8 (36.7–52.9)	41.9 (38.6–45.1)	44.5 (38.3–50.7)	39.5 (35.1–43.9)	46.1 (38.1–54.1)
Neutral	21.2 (17.4–25.0)	25.1 (18.4–31.8)	19.9 (14.7–25.1)	18.2 (8.4–28.0)	19.4 (15.5–23.3)	23.9 (16.7–31.1)	18.8 (13.6–24.0)	13.9 (3.8–24.0)
Disagree	16.5 (12.6–20.4)	16.7 (9.6–23.8)	17.9 (12.6–23.2)	10.7 (4.6–20.9)	12.7 (8.7–16.7)	11.8 (4.0–19.6)	13.7 (8.4–19.0)	11.5 (1.2–21.8)
Not applicable	24.2 (20.5–27.9)	22.3 (15.5–29.1)	24.8 (19.7–29.8)	26.2 (16.9–35.5)	25.9 (22.2–29.6)	19.8 (16.5–23.1)	28.1 (25.5–30.7)	28.5 (19.3–37.2)

Values are percentage with 95% confidence interval

Paramedic care (physical therapist, dietician, social worker, psychologist) was cancelled more often than hospital care or 1^st^ line care (general practitioner) in both Belgium and the Netherlands (**supplemental Table** **7**).

### Productivity loss

In total, 5% of the respondents in Belgium and 4% in the Netherlands reported that they lost their job due to COVID-19 (Table 7). In Belgium higher productivity losses were reported for people who were employed compared to the Netherlands. More Belgian than Dutch respondents indicated that they were absent from work for at least 1 day (30% versus 19%). The mean value of lost production among respondents in paid profession per person per week including absenteeism and presenteeism was €161.39 for Belgium and €82.69 for the Netherlands.

**Table 7:** Productivity losses among respondents in paid profession Belgium

	Belgium	the Netherlands
N	1019	811
Lost job due to COVID-19 (%)	5.1	4.4
N	1080	1247
In paid profession (%)	52	61
Worried about losing profession (%)		
Somewhat to extremely	39	40
Not at all to slightly	61	60
Weekly work hours before COVID-19	34.4 (0.31.6–37.2)	30.6 (28.0–33.2)
Weekly work hours during COVID-19	26.9 (24.3–29.5)	26.6 (24.1–29.1)
Experienced absenteeism (%)		
Some days	16.5	9.6
All days	19.2	9.1
*Absenteeism (hours/week)	1.6 (0.1–2.3)	1.3 (0.1–1.9)
*Cost absenteeism (person/week)	€133.86	€63.58
Experienced presenteeism (%)	29.5	33.7
Days experiencing presenteeism	13.3 (11.3–15.3)	9.6 (8.0–11.2)
Presenteeism (% of normal work per day)	61%	66%
Cost presenteeism (person/week)	€27.53	€19.11

Values are mean and standard deviation or percentage with 95% confidence interval

*Combined absenteeism for some days and all days

Subgroup analyses for age groups, parent status and COVID-19 infection are reported in supplemental Tables 1–9.

## Discussion

This study described the stress and concerns and quality of life access to healthcare and cancelled/delayed healthcare productivity losses during the first 8 weeks of the coronavirus lockdown in Belgium and the Netherlands. Our study indicates that the respondents in Belgium and the Netherlands underwent considerable levels of stress and concerns and experienced cancelled/postponed care and productivity loss during the COVID-19 pandemic.

When comparing results between Belgium and the Netherlands, the respondents in Belgium were more worried than respondents in the Netherlands. Reported quality of life was lower in Belgium before and during the coronavirus lockdown. Additionally, both countries experienced similar levels of cancelled or postponed care as reported by the respondents. However, the respondents report different types of cancelled specialist care. For productivity loss, both countries reported similar levels of people losing their job due to COVID-19 and worries about losing your job. However, more people experienced productivity loss in Belgium compared to the Netherlands. This results in substantial higher costs related to presenteeism and absenteeism costs in Belgium than in the Netherlands. This could be due to temporarily installed paid unemployment leave regulations in Belgium.

The global scientific body of evidence is growing for stress, concerns, reduced quality of life, and productivity loss in relation to COVID-19. Several cross-sectional studies from varies countries assessed stress, quality of life, worries and various other questions related to the impact of COVID-19 with similar questionnaires [16, 17], particularly in China [18–20]. A Chinese cross-sectional study reported that Chinese respondents have been greatly impacted in terms of non-work-related travel, work related travel, family’s daily routine, job and finance as well as reporting high levels of anxiety [18]. On the other hand, another small Chinese study among highly educated participants reported mild stressful impact scores, however, 52% of that population felt horrified and apprehensive due to the pandemic [19]. The majority of participants (58–78%) received increased support from friends and family members, increased shared feeling and caring with family members and others. A small study in Israel reported high levels of perceived stress and corona-related worries, but low levels of anxiety [16]. Female sex, younger age, corona-related loneliness, and pre-existing chronic illness were all related to higher levels of psychological distress and lower levels of quality of life.

Another Chinese survey among university students, healthcare workers and business people indicated that during a 2-week period in February 2020, the emotional state, anxiety and behavior of participants in Hubei was lower compared to reference values in February 2019, particularly for sleep quality [20]. These results from various countries underline the burden on our societies. Health education should be considered combined with psychological counseling for vulnerable individuals to cope with future outbreaks.

### Public health implications

The information gathered in this study gives a broad overview of the Belgian and Dutch society in the light of the COVID-19 pandemic and may be valuable for future health technology assessment studies in the field of COVID-19. It would be worthwhile to conduct a similar study at a later time in the pandemic, to investigate whether and how concerns, quality of life, access to care and productivity develop over time.

The current study highlights concerns, quality of life, and risk factors for psychological distress in light of the corona pandemic. More research is needed in order to fully understand the scope and correlates of societal difficulties during these challenging times. This study can be used as baseline study to assess the impact of future lockdowns in Europe.

### Strengths and limitations

Our study has some strengths and limitations. Strengths were the large representative sample that could be reached in a relatively short period of time, which was vital since the COVID-19 situation develops rapidly. We used validated questionnaires that strengthen the standardized data collection. Our population yielded a utility value of 0.85 for the entire population of the Netherlands, which is close to the reference value for the general Dutch population 0.87 [13]. This implies that our sample is comparable to a general population sample.

A limitation of this study is that there was no ability to add detailed instructions to the questionnaire as only limited instructions could be provided at the beginning of the questionnaire. To obtain a broad view of society, the questionnaire was relatively long (mean duration 15 min) and the iPCQ and iMCQ can be considered quite complex. Some misunderstanding or mis categorization can therefore not be excluded. A second limitation is that the recall period in several sections of the questionnaire is relatively long (~ 8 weeks). These responses maybe influenced by recall bias. The questions about the time before the lockdown relied on memory and were answered in a cross-sectional fashion. This might be influenced by external factors and might not represent normal life. Furthermore, the questionnaire was performed online requiring a modest digital understanding to access the questionnaire and thereby most likely limiting our sample to respondents with a higher level of digital understanding than the general population. Lastly, the sample was slightly underrepresented for the lowest educational class for Belgium.

## Conclusion

This study measured stress, quality of life, medical resource use and productivity losses in the general population in Belgium and the Netherlands during the first 8 weeks of the coronavirus lockdown. The results underline the burden on society in terms of stress, concerns, general healthcare and productivity.

## Supplementary Information

**Additional file 1: Supplemental Table 1.** Stress and concerns financials during the first 8 weeks of the national COVID-19 measures in Belgium. **Supplemental Table 2.** Stress and concerns about financials during the during the first 8 weeks of the national COVID-19 measures in the Netherlands. **Supplemental Table 3.** Concerns about care during first 8 weeks of the national COVID-19 measures in Belgium. **Supplemental Table 4.** Concerns about care during the first 8 weeks of the national COVID-19 national measures in the Netherlands. **Supplemental Table 5.** Self-perceived health and quality of life before and during the national COVID-19 measures stratified by age and COVID-19 in Belgium. **Supplemental Table 6.** Self-perceived health and quality of life before and during the national COVID-19 measures stratified by age and COVID-19 in the Netherlands. **Supplemental Table 7.** Cancelled or postponed healthcare appointments. **Supplemental Table 8.** Productivity losses in respondents in paid profession Belgium. **Supplemental Table 9.** Productivity losses among respondents in paid profession Netherlands.

## Data Availability

The datasets used and/or analyzed during the current study are available from the corresponding author on reasonable request.

## Abbreviations

EQ-5D: The EuroQol 5 Dimensions questionnaire measures health related quality of life on 5 dimensions
iMCQ: Medical Consumption Questionnaire
iPCQ: Productivity Cost Questionnaire
