# Supplementary material for: Concerns, quality of life, access to care and productivity of the general population during the first 8 weeks of the coronavirus lockdown in Belgium and the Netherlands
Source: BMC Health Serv Res. 2021 Mar 12;21:227. doi: 10.1186/s12913-021-06240-7 (PMC7953179; doi:10.1186/s12913-021-06240-7)
Supplement: Supplementary file 1 — Additional file 1: Supplemental Table 1. Stress and concerns financials during the first 8 weeks of the national COVID-19 measures in Belgium. Supplemental Table 2. Stress and concerns about financials during the during the first 8 weeks of the national COVID-19 measures in the Netherlands. Supplemental Table 3. Concerns about care during first 8 weeks of the national COVID-19 measures in Belgium. Supplemental Table 4. Concerns about care during the first 8 weeks of the national COVID-19 national measures in the Netherlands. Supplemental Table 5. Self-perceived health and quality of life before and during the national COVID-19 measures stratified by age and COVID-19 in Belgium. Supplemental Table 6. Self-perceived health and quality of life before and during the national COVID-19 measures stratified by age and COVID-19 in the Netherlands. Supplemental Table 7. Cancelled or postponed healthcare appointments. Supplemental Table 8. Productivity losses in respondents in paid profession Belgium. Supplemental Table 9. Productivity losses among respondents in paid profession Netherlands. [file 12913_2021_6240_MOESM1_ESM.docx]

**Supplemental table 1**: Stress and concerns financials during the first 8 weeks of the national COVID-19 measures in Belgium

|  | **Age** | | | **Education** | | | | | **(expected) COVID-19** | | **Children below 12 y** | | | |
| --- | --- | --- | --- | --- | --- | --- | --- | --- | --- | --- | --- | --- | --- | --- |
|  | **18-35 year** | **35-66 year** | **≥ 66 year** | **Low** | | **Middle** | | **High** | **Yes/maybe** | **No** | **Yes** | | **No** | |
| N | 641 | 1134 | 324 | 608 | | 1065 | | 426 | 564 | 1535 | 325 | | 1774 | |
| **Stress** |  |  |  |  | |  | |  |  |  |  | |  | |
| Somewhat/extremely | 35 (31.3-38.7) | 26 (23.4-28.6) | 14 (10.2-17.8) | 28 (24.4-31.6) | 23 (20.5-25.5) | | 23 (19.0-27.0) | | 31 (27.2-34.8) | 25 (22.8-27.2) | 25 (20.3-29.7) | 25 (23.0-27.0) | |  |
| Not at all to slightly | 65 (61.3-68.7) | 74 (71.4-76.6) | 86 (82.2-89.8) | 72 (68.4-75.6) | | 77 (74.5-79.5) | | 77 (73.0-81.0) | 69 (65.2-72.8) | 75 (72.8-77.2) | 75 (70.3-79.7) | | 75 (73.0-77.0) | |
| **Worried about current finances** | |  |  |  | |  | |  |  |  |  | |  | |
| Somewhat/extremely | 67 (63.4-70.6) | 58 (56.1-61.9) | 40 (34.7-45.3) | 64 (60.2-67.8) | | 58 (55.0-61.0) | | 54 (49.3-58.7) | 65 (61.1-68.9) | 57 (54.5-59.5) | 67 (61.9-72.1) | | 57 (54.7-59.3) | |
| Not at all to slightly | 33 (29.4-36.6) | 42 (39.1-44.9) | 60 (54.7-65.3) | 36 (32.2-39.8) | | 42 (39.0-45.0) | | 46 (41.3-50.7) | 35 (31.1-38.9) | 43 (40.5-45.5) | 33 (27.9-38.1) | | 43 (40.7-45.3) | |
| **Worried about future finances** | |  |  |  | |  | |  |  |  |  | |  | |
| Somewhat/extremely | 69 (65.4-72.6) | 68 (65.3-70.7) | 50 (44.6-55.4) | 68 (64.3-71.7) | | 66 (63.2-68.8) | | 62 (57.4-66.6) | 71 (67.3-74.7) | 63 (60.6-65.4) | 75 (70.3-79.7) | | 64 (61.8-66.2) | |
| Not at all to slightly | 31 (27.4-34.6) | 32 (29.3-34.7) | 50 (44.6-55.4) | 32 (28.3-35.7) | | 34 (31.2-36.8) | | 38 (33.4-42.6) | 29 (25.3-32.7) | 37 (34.6-39.4) | 25 (20.3-29.7) | | 36 (33.8-38.2) | |
| **Worried about national economy** | |  |  |  | |  | |  |  |  |  | |  | |
| Somewhat/extremely | 83 (80.1-85.9) | 89 (87.2-90.8) | 92 (89.0-95.0) | 86 (83.2-88.8) | | 88 (86.0-90.0) | | 89 (86.0-92.0) | 89 (86.4-91.6) | 87 (85.3-88.7) | 87 (83.3-90.7) | | 87 (85.4-88.6) | |
| Not at all to slightly | 17 (14.1-19.9) | 11 (9.2-12.8) | 8 (5.0-11.0) | 14 (11.2-16.8) | | 12 (10.0-14.0) | | 11 (8.0-14.0) | 11 (8.4-13.6) | 13 (11.3-14.7) | 13 (9.3-16.7) | | 13 (11.4-14.6) | |

Values are percentage with 95% confidence interval

**Supplemental table 2:** Stress and concerns about financials during the during the first 8 weeks of the national COVID-19 measures in the Netherlands

|  | **Age** | | | **Education** | | | **(expected) COVID-19** | | **Children below 12 y** | |
| --- | --- | --- | --- | --- | --- | --- | --- | --- | --- | --- |
|  | **18-35** | **35-66** | **≥ 67 year** | **Low** | **Middle** | **High** | **Yes/maybe** | **No** | **Yes** | **No** |
| N | 560 | 1175 | 323 | 526 | 588 | 944 | 507 | 1551 | 454 | 1604 |
| **Stress** |  |  |  |  |  |  |  |  |  |  |
| Somewhat/extremely | 20 (16.7-23.3) | 14 (12.0-16.0) | 5 (2.6-7.4) | 12 (9.2-14.8) | 15 (12.1-17.9) | 15 (12.7-17.3) | 17 (0.13.7-20.3) | 13 (11.3-14.7) | 17 (13.5-20.5) | 14 (12.3-15.7) |
| Not at all to slightly | 80 (76.7-83.3) | 86 (84.0-88.0) | 95 (92.6-97.4) | 88 (85.2-90.8) | 85 (82.1-87.9) | 85 (82.7-87.3) | 83 (79.7-86.2) | 87 (85.3-88.7) | 83 (79.5-86.5) | 86 (84.3-87.7 |
| **Worried about current financials** | |  |  |  |  |  |  |  |  |  |
| Somewhat/extremely | 67 (63.1-70.9) | 45 (42.2-47.8) | 58 (52.6-63.4) | 42 (37.8-46.2) | 48 (44.0-52.0) | 52 (48.8-55.2) | 61 (56.8-65.2) | 44 (41.5-46.5) | 66 (61.6-70.4) | 43 (40.6-45.4) |
| Not at all to slightly | 33 (29.1-36.9) | 55 (52.2-57.8) | 42 (36.6-47.4) | 58 (53.8-62.2) | 52 (48.0-56.0) | 48 (44.8-51.2) | 39 (34.8-43.2) | 56 (53.5-58.5) | 34 (29.6-38.4)) | 57 (54.6-59.4) |
| **Worried about future financials** | |  |  |  |  |  |  |  |  |  |
| Somewhat/extremely | 66 (62.1-69.9) | 55 (52.2-57.8) | 46 (40.6-51.4) | 53 (48.7-57.3) | 57 (53.0-61.0) | 60 (56.9-63.1) | 63 (58.8-67.2) | 52 (49.5-54.5) | 71 (66.8-75.2) | 53 (50.6-55.4) |
| Not at all to slightly | 34 (30.1-37.9) | 45 (42.2-47.8) | 54 (48.6-59.4) | 47 (42.7-51.3) | 43 (39.0-47.0) | 40 (36.9-43.1) | 37 (32.8-41.2) | 48 (45.5-50.5) | 39 (34.5-43.5) | 47 (44.6-49.4) |
| **Worried about national economy** | |  |  |  |  |  |  |  |  |  |
| Somewhat/extremely | 85 (82.0-88.0) | 86 (84.0-88.0) | 91 (87.9-94.1) | 85 (81.9-88.1) | 87 (84.3-89.7) | 87 (84.9-89.1) | 92 (89.6-94.4) | 85 (83.2-86.8) | 86 (82.8-89.2) | 87 (85.3-88.6) |
| Not at all to slightly | 15 (11.1-16.9) | 14 (12.0-16.0) | 9 (5.9-12.1) | 15 (11.9-18.1) | 13 (10.3-15.7) | 13 (10.9-15.1) | 8 (5.6-10.4) | 15 (13.2-16.8) | 14 (10.8-17.2) | 13 (11.3-14.6) |

Values are percentage with 95% confidence interval

**Supplemental table 3:** Concerns about care during first 8 weeks of the national COVID-19 measures in Belgium

|  | **Age (year)** | | | **Education** | | | **(expected) COVID-19** | | **Children below 12 y** | |
| --- | --- | --- | --- | --- | --- | --- | --- | --- | --- | --- |
|  | **18-35** | **35-65** | **≥ 66 year** | **Low** | **Middle** | **High** | **Yes/maybe** | **No** | **Yes** | **No** |
| N | 227 | 646 | 253 | 389 | 653 | 223 | 347 | 918 | 156 | 1109 |
| **Availability of medication** | |  |  |  |  |  |  |  |  |  |
| Somewhat/extremely | 35 (28.8-41.2) | 33 (29.4-36.6) | 27 (21.5-32.4) | 33 (28.3-37.7) | 34 (30.4-37.6) | 27 (21.2-32.8) | 39 (33.9-44.1) | 30 (27.0-33.0) | 44 (36.2-51.8) | 30 (27.3-32.7) |
| Not at all to slightly | 65 (58.8-71.2) | 67 (63.4-70.6) | 73 (67.5-78.5) | 6762.3-71.7) | 66 (62.4-69.6) | 73 (67.2-78.8) | 61 (55.9-66.1) | 70 (67.0-73.0) | 56 (48.2-63.8) | 70 (67.3-72.7) |
|  |  |  |  |  |  |  |  |  |  |  |
| N | 103 | 279 | 89 | 144 | 249 | 78 | 161 | 310 | 70 | 401 |
| **Medical condition** |  |  |  |  |  |  |  |  |  |  |
| Somewhat/extremely | 62 (55.7-68.3) | 55 (51.2-58.2) | 38 (27.9-48.1) | 53 (44.8-61.2) | 53 (46.8-59.2) | 55 (44.0-66.0) | 63 (55.5-70.5) | 49 (43.4-54.6) | 63 (51.7-74.3) | 52 (47.1-56.9) |
| Not at all to slightly | 38 (31.7-44.3) | 45 (41.2-48.8) | 62 (51.9-72.1) | 47 (38.8-55.2) | 47 (40.8-53.2) | 45 (34.0-56.0) | 37 (29.5-44.5) | 51 (0.45-0.57) | 37 (25.7-48.3) | 48 (43.1-52.9) |
|  |  |  |  |  |  |  |  |  |  |  |
| N | 641 | 1134 | 324 | 608 | 1065 | 426 | 564 | 1535 | 325 | 1774 |
| **Access to care** |  |  |  |  |  |  |  |  |  |  |
| Somewhat/extremely | 56 (49.5-62.5) | 54 (50.2-57.8) | 32 (26.9-37.1) | 56 (52.1-60.0) | 59 (56.0-61.9) | 49 (44.3-53.7) | 59 (54.9-63.1) | 50 (47.5-52.5) | 62 (56.7-67.3) | 51 (48.7-53.3) |
| Not at all to slightly | 44 (37.5-50.5) | 46 (42.2-49.8) | 58 (52.6-63.4) | 44 (40.1-47.9) | 41 (38.0-44.0) | 51 (46.3-55.7) | 41 (36.9-45.1) | 50 (47.5-52.5) | 38 (32.7-43.3) | 49 (46.7-51.3) |

Values are percentage with 95% confidence interval

**Supplemental table 4:** Concerns about care during the first 8 weeks of the national COVID-19 national measures in the Netherlands

|  | **Age (year)** | | | **Education** | | | **(expected) COVID-19** | | **Children below 12 y** | |  |
| --- | --- | --- | --- | --- | --- | --- | --- | --- | --- | --- | --- |
|  | **18-35** | **35-65** | **≥ 67** | **Low** | **Middle** | **High** | **Yes/maybe** | **No** | **Yes** | **No** | |
| N | 227 | 646 | 253 | 347 | 327 | 452 | 298 | 828 | 199 | 927 | |
| **Availability of medication** | | | |  | | |  | |  | |  |
| Somewhat/extremely | 57 (50.6-63.4) | 27 (23.6-30.4) | 24 (18.7-29.3) | 30 (25.2-34.8) | 26 (21.2-30.8) | 38 (33.5-42.5) | 49 (43.3-54.7) | 26 (23.0-29.0) | 64 (57.3-70.7) | 16 (13.6-18.4) | |
| Not at all/slightly | 43 (36.6-49.4) | 73 (69.6-76.4) | 76 (70.7-81.3) | 70 (65.2-74.8) | 74 (69.2-78.8) | 62 (57.5-66.5) | 51 (45.3-56.7) | 74 (71.0-77.0) | 36 (29.3-42.7) | 84 (81.6-86.4) | |
|  |  |  |  |  |  |  |  |  |  |  | |
| N | 121 | 219 | 60 | 104 | 109 | 187 | 131 | 269 | 111 | 289 | |
| **Medical condition** | | | |  | | |  | |  | |  |
| Somewhat/extremely | 84 (77.5-90.5) | 58 (51.4-64.5) | 38 (25.7-50.3) | 58 (48.5-67.5) | 64 (55.0-73.0) | 71 (64.5-77.5) | 81 (74.3-87.8) | 55 (49.1-60.9) | 84 (77.2-90.8) | 55 (49.3-60.7) | |
| Not at all/slightly | 16 (94.7-22.5) | 42 (35.45-48.5) | 62 (49.7-74.3) | 42 (32.5-51.5) | 36 (27.0-45.0) | 29 (022.5-35.5) | 19 (12.3-25.7) | 45 (39.1-50.9) | 16 (9.2-22.8) | 45 (39.3-50.7) | |
|  |  |  |  |  |  |  |  |  |  |  | |
| N | 560 | 1175 | 323 | 526 | 588 | 944 | 507 | 1551 | 454 | 1604 | |
| **Access to care** | | | |  | | |  | |  | |  |
| Somewhat/extremely | 64 (60.0-68.0) | 47 (44.1-49.9) | 56 (50.9-61.4) | 45 (40.7-49.3) | 51 (47.0-55.0) | 54 (50.8-57.2) | 65 (60.8-69.2) | 46 (43.5-48.5) | 69 (64.7-73.3) | 46 (43.6-48.4) | |
| Not at all/slightly | 36 (32.0-40.0) | 53 (50.1-55.9) | 44 (38.6-49.4) | 55 (50.7-59.3) | 49 (45.0-53.0) | 46 (42.8-49.2) | 35 (30.8-39.2) | 54 (51.5-56.5) | 31 (26.7-35.3) | 54 (51.5-56.4) | |

Values are percentage with 95% confidence interval

**Supplemental table 5:** Self-perceived health and quality of life before and during the national COVID-19 measures stratified by age and COVID-19 in Belgium

|  | **18-35 year** | **35-65 year** | **≥ 66 year** | **(expected)**  **COVID-19** | **No COVID-19** |
| --- | --- | --- | --- | --- | --- |
| N | 641 | 1134 | 324 | 534 | 1432 |
| Perceived health during COVID-19 | 75.7 (72.4-79.0) | 71.1 (68.4-73.6) | 73.4 (68.6-78.2) | 71.5 (67.7-75.3) | 73.3 (71.0-75.6) |
| Perceived health before COVID-19 | 77.7 (74.4-80.9) | 72.6 (70.0-75.2) | 74.8 (70.0-79.5) | 74.0 (70.3-77.7) | 74.7 (72.4-82.1) |
| EQ-5D during COVID-19 measures | 0.81 (0.78-0.84) | 0.78 (0.76-0.80) | 0.80 (0.76-0.84) | 0.78 (0.74-0.82) | 0.80 (0.78-0.82) |
| EQ-5D before COVID-19 measures | 0.84 (0.81-0.86) | 0.80 (0.78-0.82) | 0.82 (0.78-0.86) | 0.81 (0.78-0.84) | 0.82 (0.80-0.84) |

Values are mean and 95% confidence interval

**Supplemental table 6:** Self-perceived health and quality of life before and during the national COVID-19 measures stratified by age and COVID-19 in the Netherlands

|  | **18-35 year** | **35-66 year** | **≥ 67 year** | **(expected)**  **COVID-19** | **No COVID-19** |
| --- | --- | --- | --- | --- | --- |
| N | 560 | 1175 | 323 | 411 | 1466 |
| Perceived health during COVID-19 | 76.2 (72.7-79.7) | 71.2 (68.6-73.8) | 74.3 (69.5-79.1) | 72.9 (68.6-77.2) | 73.1 (70.8-75.4) |
| Perceived health before COVID-19 | 77.7 (74.3-81.1) | 73.2 (70.7-75.7) | 75.6 (70.9-80.2) | 74.9 (70.7-79.1) | 74.8 (72.6-77.0) |
| EQ-5D during COVID-19 measures | 0.84 (0.81-0.87) | 0.83 (0.81-0.85) | 0.84 (0.80-0.88) | 0.82 (0.78-0.86) | 0.84 (82.1-85.9) |
| EQ-5D before COVID-19 measures | 0.87 (0.84-0.90) | 0.85 (0.83-0.87) | 0.86 (0.82-0.90) | 0.85 (0.82-0.88) | 0.86 (0.84-0.88) |

Values are mean and 95% confidence interval

**Supplemental table 7:** Cancelled or postponed healthcare appointments

|  | **Belgium** | | | | **Netherlands** | | | |
| --- | --- | --- | --- | --- | --- | --- | --- | --- |
|  | Total | 18- 35 year | 35-65 year | ≥ 66 year | Total | 18-35 year | 35-66 year | ≥ 67 year |
| N | 2099 | 641 | 1134 | 324 | 2058 | 560 | 1175 | 323 |
|  |  |  |  |  |  |  |  |  |
| Total cancelled care (N/%) | 443 (21.1) | 163 (25.4) | 211 (18.6) | 54 (16.6) | 500 (24.3) | 144 (25.7) | 266 (22.6) | 52 (16.1) |
| 1e line care (GP/NP/OP) | 5.3 (4.3-6.3) | 9.9 (7.6-12.2) | 2.9 (1.9-3.9) | 1.3 (0.6-2.5) | 6.3 (5.3-7.3) | 8.1 (5.8-10.4) | 5.1 (3.8-6.4) | 1.7 (0.3-3.1) |
| 2e line care - hospital care | 7.0 (5.9-8.1) | 7.2 (5.2-9.2) | 6.6 (5.2-8.0) | 6.7(4.0-9.4) | 7.6 (6.5-8.7) | 7.6 (5.4-9.8) | 7.2 (5.7-8.7) | 6.5 (3.8-9.2) |
| Other paramedic care | 8.8 (7.6-10.0) | 8.3 (6.2-10.4) | 9.1 (7.4-10.8) | 8.6 (5.5-11.7) | 10.4 (9.1-11.7) | 10.0 (7.5-12.5) | 10.3 (8.6-12.0) | 7.9 (5.0-10.8) |

Values are mean and 95% confidence interval; GP: general practitioner, NP: nurse practitioner, OP: occupational physician, hospital care includes (emergency, outpatient + inpatient clinic), paramedic care (physical therapist, dietician, social worker, psychologist)

**Supplemental table 8:** Productivity losses in respondents in paid profession Belgium

|  | **Age (year)** | | | **Education** | | | **(expected) COVID-19** | | **Children below 12 y** | |
| --- | --- | --- | --- | --- | --- | --- | --- | --- | --- | --- |
|  | **18-35** | **35-65** | **≥ 66** | **Low** | **Middle** | **High** | **Yes** | **No** | **Yes** | **No** |
| N | 265 | 445 | 309 | 359 | 531 | 129 | 244 | 775 | 72 | 947 |
| Lost job due to COVID-19 (%) | 14.3 | 2.7 | 0.6 | 5.6 | 4.7 | 5.4 | 4.1 | 5.4 | 15.3 | 4.3 |
|  |  |  |  |  |  |  |  |  |  |  |
| N | **376** | **689** | **15** | **249** | **534** | **297** | **320** | **760** | **253** | **827** |
| In paid profession | 59 | 61 | 5 | 41 | 50 | 70 | 57 | 50 | 78 | 46 |
| Worried about losing profession (%) |  |  |  |  |  |  |  |  |  |  |
| Somewhat/extremely | 44 | 37 | 20 | 41 | 38 | 40 | 44 | 37 | 46 | 37 |
| Not at all to slightly | 56 | 63 | 80 | 59 | 62 | 60 | 56 | 63 | 54 | 63 |
| Weekly work hours before COVID | 33.9 (29.1-38.7) | 35.0 (31.4-38.6) | 22.6 (17.9-27.3) | 32.9 (27.1-38.7) | 34.2 (30.2-38.2) | 36.2 (30.7-41.7) | 34.1 (28.9-39.3) | 34.6 (31.2-38.0) | 34.2 (28.4-40.0) | 34.5 (31.2-37.7) |
| Weekly work hours during COVID | 26.7 (22.2-31.2) | 27.3 (24.0-30.6) | 9.3 (0-24.0) | 24.0 (18.7-29.3) | 26.1 (22.4-29.8) | 30.6 (25.4-35.8) | 26.3 (21.5-31.1) | 27.1 (23.9-30.3) | 27.1 (21.6-32.6) | 27.1 (24.1-30.1) |
| Experienced absenteeism (%) |  |  |  |  |  |  |  |  |  |  |
| Some days | 15.7 | 16.8 | 20.0 | 17.7 | 15.5 | 17.2 | 22.2 | 14.1 | 18.2 | 16.0 |
| All days | 20.7 | 18.4 | 13.3 | 24.1 | 20.6 | 12.5 | 21.9 | 18.0 | 16.2 | 20.1 |
| Absenteeism: (hours/week) | 1.6±0.8 | 1.5±0.8 | 1.5±0.7 | 1.7±0.8 | 1.6±0.8 | 1.4±0.7 | 1.7±0.8 | 1.5±0.8 | 1.5±0.8 | 1.6±0.8 |
| Costs absenteeism (person/wk) | €168.07 | €159.83 | €5.69 | €132.68 | €144.01 | €128.97 | €173.09 | €125.76 | €176.17 | €131.48 |
| Experienced presenteeism | 33.5 | 27.9 | 6.7 | 27.3 | 27.7 | 34.7 | 45.9 | 22.6 | 34.0 | 28.2 |
| Days experiencing presenteeism | 11.9±13.6 | 14.3±15.1 | 4.0±na | 13.6±15.8 | 12.8±14.9 | 14.0±13.1 | 12.6±14.4 | 14.0±14.6 | 14.0±14.4 | 13.2±14.6 |
| Presenteeism (% normal work/d) | 63% | 60% | 50% | 63% | 60% | 61% | 59% | 63% | 62% | 61% |
| Cost presenteeism (person/wk) | €29.81 | €34.49 | €0.14 | €18.82 | €24.68 | €48.55 | €46.56 | €20.36 | €48.88 | €23.63 |

Values are mean and standard deviation or percentage with 95% confidence interval

**Supplemental table 9:** Productivity losses among respondents in paid profession Netherlands

|  | **Age** | | | **Education** | | | **(expected) COVID-19** | | **Children below 12 y** | |
| --- | --- | --- | --- | --- | --- | --- | --- | --- | --- | --- |
|  | **18-35** | **35-66** | **≥67** | **Low** | **Middle** | **High** | **Yes** | **No** | **Yes** | **No** |
| N | 101 | 413 | 297 | 360 | 241 | 210 | 146 | 665 | 38 | 773 |
| Lost job due to COVID-19 (%) | 12.9 | 5.6 | 0.0 (0.0-0.0) | 2.5 | 4.6 | 7.6 | 11.6 | 2.9 | 13.2 | 4.0 |
|  |  |  |  |  |  |  |  |  |  |  |
| N | **459** | **762** | **26** | **166** | **347** | **734** | **361** | **886** | **416** | **831** |
| In paid profession (%) | 82 | 65 | 8 | 32 | 59 | 78 | 71 | 57 | 92 | 52 |
| Worried about losing profession (%) |  |  |  |  |  |  |  |  |  |  |
| Somewhat/extremely | 40 | 31 | 31 | 22 | 38 | 44 | 48 | 42 | 59 | 31 |
| Not at all/slightly | 60 | 69 | 69 | 78 | 62 | 56 | 52 | 58 | 41 | 69 |
| Weekly work hours before COVID | 28.5 (24.4-32.6) | 32.1 (28.8-35.4) | 22.1 (6.2-38.0) | 26.6 (19.9-33.3) | 29.1 (24.3-33.9) | 32.2 (28.8-35.6) | 30.3 (25.6-35.0) | 30.7 (27.7-33.7) | 31.0 (26.6-35.4) | 31.0 (27.9-34.1) |
| Weekly work hours during COVID | 24.1 (20.2-28.0) | 28.4 (25.2-31.6) | 14.5 (1.0-28.0) | 22.6 (16.2-29.0) | 25.4 (20.8-30.0) | 28.0 (24.8-31.2) | 26.8 (22.2-31.4) | 26.4 (23.5-29.3) | 27.1 (22.8-31.4) | 26.3 (23.3-29.3) |
| Experienced absenteeism (%) |  |  |  |  |  |  |  |  |  |  |
| Some days | 8.9 | 10.0 | 11.5 | 11.4 | 11.2 | 8.4 | 14.1 | 7.8 | 7.5 | 10.7 |
| All days | 9.4 | 8.4 | 23.1 | 11.4 | 10.1 | 8.0 | 8.3 | 9.4 | 6.5 | 10.3 |
| Absenteeism (mean hours/wk) | 1.3±0.6 | 1.3±0.5 | 1.6±0.9 | 1.3±0.6 | 1.3±0.6 | 1.3±0.6 | 1.3±0.6 | 1.3±0.6 | 1.2±0.5 | 1.3±0.7 |
| Costs absenteeism (person/wk) | €82.76 | €65.97 | €15.37 | €36.12 | €65.38 | €75.49 | €67.71 | €62.01 | €69.55 | €61.71 |
| Experienced presenteeism (%) | 40.7 | 30.1 | 15.4 | 26.5 | 29.7 | 37.2 | 43.8 | 29.6 | 42.3 | 28.9 |
| Days of experiencing presenteeism | 9±10.6 | 10.2±12.2 | 3.8±0.5 | 8.3±12.9 | 9.0±10.4 | 10.0±11.6 | 10.7±0.7 | 9.4±10.7 | 10.2±11.3 | 9.2±11.6 |
| Presenteeism: (% of normal work/d) | 69% | 65% | 58% | 59% | 67% | 68% | 69% | 66% | 66% | 67% |
| Costs presenteeism (person/wk) | €24.86 | €20.97 | €0.41 | €7.10 | €14.19 | €27.94 | €28.69 | €15.53 | €39.02 | €13.20 |

Values are mean and standard deviation or percentage with 95% confidence intervals
